# Supplementary material for: 3-Chymotrypsin-like Protease (3CLpro) of SARS-CoV-2: Validation as a Molecular Target, Proposal of a Novel Catalytic Mechanism, and Inhibitors in Preclinical and Clinical Trials
Source: Viruses. 2024 May 24;16(6):844. doi: 10.3390/v16060844 (PMC11209289; doi:10.3390/v16060844)
Supplement: Supplementary file 1 [file viruses-16-00844-s001.zip › viruses-3017139-supplementary.pdf]

Supporting information

3-Chymotrypsin-like Protease (3CLpro) of SARS-CoV-2: Validation as a Molecular Target, Proposal of a Novel Catalytic Mechanism, and Inhibitors in Preclinical and Clinical Trials

Vitor Martins de Freitas Amorim[1,], Eduardo Pereira Soares[1], Anielle Salviano de Almeida Ferrari[1], Davi Gabriel Salustiano Merighi[1], Robson Francisco de Souza[1], Cristiane Rodrigues Guzzo[1,*], Anacleto Silva de Souza[1,*]

[1]Department of Microbiology, Institute of Biomedical Sciences, University of São Paulo, São Paulo, Brazil.

* Corresponding author
E-mail: crisguzzo@usp.br and crisguzzo@gmail.com
E-mail: anacletosilvadesouza@usp.br and anacletosilvadesouza@gmail.com

Running title: main protease and drugs in clinical trials

To whom correspondence should be addressed:

Cristiane R. Guzzo, Ph.D, Department of Microbiology, Institute of Biomedical Sciences, University of São Paulo, Av. Prof. Lineu Prestes, 1374, Cidade Universitária, 5508-000, São Paulo/SP, Brazil, +55 11 3091-7298; E-mail: crisguzzo@usp.br

Anacleto Silva de Souza, Ph.D, Department of Microbiology, Institute of Biomedical Sciences, University of São Paulo, Av. Prof. Lineu Prestes, 1374, Cidade Universitária, 5508-000, São Paulo/SP, Brazil, +55 11 3091-7298; E-mail: anacletosilvadesouza@usp.br

Keywords: SARS-CoV-2; 3CLpro; novel mechanism of catalysis; preclinical and clinical trials; triad.

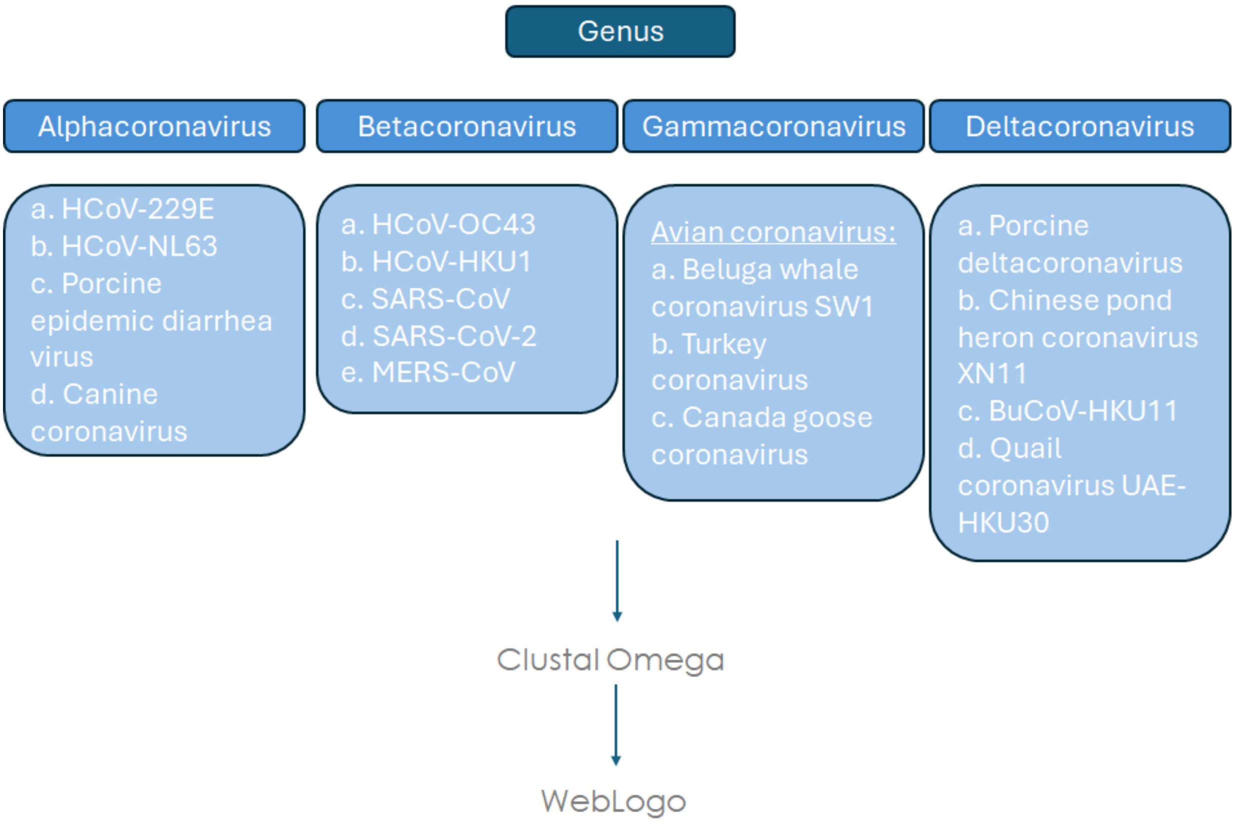

Figure S1. Workflow for identifying representative sequences of each coronavirus genus. Initially, a search was conducted using BLASTP to find representatives of each genus, excluding SARS-CoV-2 3CLpro sequences at all stages. This search resulted in sixteen sequences from different coronavirus lineages (**Figure S2**). Subsequently, a multiple sequence alignment of the amino acid sequences was performed using Clustal (https://www.ebi.ac.uk/jdispatcher/msa/clustalo) with default parameters. Finally, the alignment was submitted to the WebLogo site to generate the sequence logo (https://weblogo.berkeley.edu/logo.cgi).

```
>6W81_A Chain A, Peptidase C30 [Porcine epidemic diarrhea virus]
AGLRKMAQPSGVVEKCIVRVCYGNMALNGLWLGDTVICPRHVIASSTTSTIDYDYALSVLRLHNFSISSGNVFLGVVGVT
MRGALLQIKVNQNNVHTPKYTYRTVRPGESFNILACYDGSAAGVYGVNMRSNYTIRGSFINGACGSPGYNINNGTVEFCY
LHQLELGSGCHVGSDLDGVMYGGYEDQPTLQVEGASSLFTENVLAFLYAALINGSTWWLSSSRIAVDRFNEWAVHNGMTT
VVNTDCFSILAAKTGVDVQRLLASIQSLHKNFGGKQILGYTSLTDEFTTGEVIRQMYGVNLQ

>7XJW_A Chain A, ORF1a polyprotein [Canine coronavirus]
SGLRKMAQPSGLVEPCIVRVSYGNNVLNGLWLGDEVICPRHVIASDTTRVINYENEMSSVRLHNFSVSKNNVFLGVVSAK
YKGVNLVLKVNQVNPNTPEHKFKSIKAGESFNILACYEGCPGSVYGVNMRSQGTIKGSFIAGTCGSVGYVLENGILYFVY
MHHLELGNGSHVGSNLEGEMYGGYEDQPSMQLEGTNVMSSDNVVAFLYAALINGERWFVTNTSMSLESYNTWAKTNSFTE
LSSIDAFSMLAAKTGQSVEKLLDSIVRLNKGFGGRTILSYGSLCDEFTPTEVIRQMYGVNLQ

>3TLO_A Chain A, 3C-like proteinase [Human coronavirus NL63]
SGLKKMAQPSGCVERCVVRVCYGSTVLNGVWLGDTVTCPRHVIAPSTTVLIDYDHAYSTMRLHNFSVSHNGVFLGVVGVT
MHGSVLRIKVSQSNVHTPKHVFKTLKPGDSFNILACYEGIASGVFGVNLRTNFTIKGSFINGACGSPGYNVRNDGTVEFC
YLHQIELGSGAHVGSDFTGSVYGNFDDQPSLQVESANLMLSDNVVAFLYAALLNGCRWWLCSTRVNVDGFNEWAMANGYT
SVSSVECYSILAAKTGVSVEQLLASIQHLHEGFGGKNILGYSSLCDEFTLAEVVKQMYGVNLQ

>NP_835346.1 putative coronavirus nsp2 (3CL-PRO) [Human coronavirus 229E]
AGLRKMAQPSGFVEKCVVRVCYGNTVLNGLWLGDIVYCPRHVIASNTTSAIDYDHEYSIMRLHNFSIISGTAFLGVVGAT
```

MHGVTLKIKVSQTNMHTPRHSFRTLKSGEGFNILACYDGCAQGVFGVNMRTNWTIRGSFINGACGSPGYNLKNGEVEFVY
MHQIELGSGSHVGSSFDGVMYGGFEDQPNLQVESANQMLTVNVVAFLYAAILNGCTWWLKGEKLFVEHYNEWAQANGFTA
MNGEDAFSILAAKTGVCVERLLHAIQVLNNGFGGKQILGYSSLNDEFSINEVVKQMFGVNLQ

>SARS-CoV-2
SGFRKMAFPSGKVEGCMVQVTCGTTTLNGLWLDDVVYCPRHVICTSEDMLNPNYEDLLIRKSNHNFLVQA
GNVQLRVIGHSMQNCVLKLKVDTANPKTPKYKFVRIQPGQTFSVLACYNGSPSGVYQCAMRPNFTIKGSF
LNGSCGSVGFNIDYDCVSFCYMHHMELPTGVHAGTDLEGNFYGPFVDRQTAQAAGTDTTITVNVLAWLYA
AVINGDRWFLNRFTTTLNDFNLVAMKYNYEPLTQDHVDILGPLSAQTGIAVLDMCASLKELLQNGMNGRT
ILGSALLEDEFTPFDVVRQCSGVTFQ

>2Q6G_A Chain A, severe acute respiratory syndrome coronavirus (SARS-CoV) [SARS coronavirus BJ01]
SGFRKMAFPSGKVEGCMVQVTCGTTTLNGLWLDDTVYCPRAVICTAEDMLNPNYEDLLIRKSNHSFLVQAGNVQLRVIGH
SMQNCLLRLKVDTSNPKTPKYKFVRIQPGQTFSVLACYNGSPSGVYQCAMRPNHTIKGSFLNGSCGSVGFNIDYDCVSFC
YMHHMELPTGVHAGTDLEGKFYGPFVDRQTAQAAGTDTTITLNVLAWLYAAVINGDRWFLNRFTTTLNDFNLVAMKYNYE
PLTQDHVDILGPLSAQTGIAVLDMCAALKELLQNGMNGRTILGSTILEDEFTPFDVVRQCSGVTFQ

>7D3C_A Chain A, 3C-like proteinase [Middle East respiratory syndrome-related coronavirus]
SGLVKMSHPSGDVEACMVQVTCGSMTLNGLWLDDTVWCPRHVMCPADQLSDPNYDALLISMTNHSFSVQKHIGAPANLRV
VGHAMQGTLLKLTVDVANPSTPAYTFTTVKPGAAFSVLACYNGRPTGTFTVVMRPNYTIKGSFLCGSCGSVGYTKEGSVI
NFCYMHQMELANGTHTGSAFDGTMYGAFMDKQVHQVQLTDKYCSVNVVAWLYAAILNGCAWFVKPNRTSVVSFNEWALAN
QFTEFVGTQSVDMLAVKTGVAIEQLLYAIQQLYTGFQGKQILGSTMLEDEFTPEDVNMQIMGVVMQ

>YP_009555250.1 nsp2 [Human coronavirus OC43]
SGIVKMVNPTSKVEPCVVSVTYGNMTLNGLWLDDKVYCPRHVICSASDMTNPDYTNLLCRVTSSDFTVLFDRLSLTVMSY
QMRGCMLVLTVTLQNSRTPKYTFGVVKPGETFTVLAAYNGKPQGAFHVTMRSSYTIKGSFLCGSCGSVGYVIMGDCVKFV
YMHQLELSTGCHTGTDFNGDFYGPYKDAQVVQLLIQDYIQSVNFVAWLYAAILNNCNWFVQSDKCSVEDFNVWALSNGFS
QVKSDLVIDALASMTGVSLETLLAAIKRLKNGFQGRQIMGSCSFEDELTPSDVYQQLAGIKLQ

>3D23_A Chain A, 3C-like proteinase [Human coronavirus HKU1 (isolate N1)]
ASSGIVKMVSPTSKIEPCIVSVTYGSMTLNGLWLDDKVYCPRHVICSSSNMNEPDYSALLCRVTLGDFTIMSGRMSLTVV
SYQMQGCQLVLTVSLQNPYTPKYTFGNVKPGETFTVLAAYNGRPQGAFHVTMRSSYTIKGSFLCGSCGSVGYVLTGDSVK
FVYMHQLELSTGCHTGTDFTGNFYGPYRDAQVVQLPVKDYVQTVNVIAWLYAAILNNCAWFVQNDVCSTEDFNVWAMANG
FSQVKADLVLDALASMTGVSIETLLAAIKRLYMGFQGRQILGSCTFEDELAPSDVYQQLAGV

>8E7N_A Chain A, main protease [Beluga whale coronavirus SW1]
AGIKKMVAPSSAVEQCVVSVVHGNTQLNGLWLNDYVLCPRHILGKYTGEQWRDALINANNFDFHILYKGMELQVVGRELV
GALLKLKVSMVNANTPKYKFAKARIGDNFSIACAYNGHVSGLYTVTLRENGTLKGSFMSGSCGSVGYNVTNEGVEFVYMH
HLELPGCVHGGSDLHGIFYGGYVDEEVLQRIPPAPANSRNIVAWLYAAVYNNCDWFVKYGPKQVMSVEDFNEWASGYGFT
KFEYHLAFDVFSAATGVSVEQMLAAIKELADGWNYAPVLGSFHLDDEYSPEMIMQQTSGIVLQ

>YP_001941178.1 NSP5 [Turkey coronavirus]
AGFKKLVCPSSAVENCIVSVSYRGNNLNGLWLGDAIYCPRHVLGKFSGDQWSDVLNLANNHEFEVVTQNGVTLNVVSRRL
RGAVLILQTAVANADTPNYKFVKANCGDSFTIACSYGGTVVGLYPVTMRPNGTIRASFLAGACGSVGFNIEKGVVTFVYM
HHLELPNALHTGTDLFGDFYGDYVDEEVAQRVPPDNLVTNNIVAWLYAAIISVKESSFSLPKWLESTTVSVEDYNKWAGD
NGFTPFSTSTAITKLSAITGVDVCKLLRTIMVKSSQWGSDPILGQYNFEDELTPESVFNQVGGVRLQ

>YP_009755914.1 nsp5 [Canada goose coronavirus]
AGFKKLVAPSSGIENVLVMVRFKGNVLNGIWLNDLIYCPRHVIGKYSGDDWQNALHMANNFDFEVISNKIGGLSVIERRM
QGALLVLRVNQSNKNTPKFKFVKAHDGDTFTIACSYNGVIAGLYPCTLRANGTIKGSFGLGSCGSIGYNLVNGIYELCYM
HHLELPGAIHAGTDLSGNFYGDFVDEEKAQFVKPDPLIANNVVAWLYAAIINYRESHYCYPKWLESSSVSLEEFNAWAKD
NGFTSFIDGPVFQKLASITGVELGRVLRTILTKHACWGNDPILGSFSFDDEITPFSVVEQCGGVVLQ

>8E7C_A Chain A, Main Protease [Porcine deltacoronavirus]
AGIKILLHPSGVVERCMVSVVYNGSALNGIWLKNVVYCPRHVIGKFRGDQWTHMVSIADCRDFIVKCPIQGIQLNVQSVK
MVGALLQLTVHTNNTATPDYKFERLQPGSSMTIACAYDGIVRHVYHVVLQLNNLIYASFLNGACGSVGYTLKGKTLYLHY
MHHIEFNNKTHSGTDLEGNFYGPYVDEEVIQQQTAFQYYTDNVVAQLYAHLLTVDARPKWLAQSQISIEDFNSWAANNSF
ANFPCEQTNMSYIMGLSQTARVPVERILNTIIQLTTNRDGACIMGSYDFECDWTPEMVYNQAPISLQ

>Chinese pond heron coronavirus XN11
GIKRLLSPSGSVEKCMVSVHYRGLTLNGIWLNNVIYCPRHILGKYQASFWQDAVKVADTRDFVINSQHSKIQFRPVGLRLNNA
ILQIVLPTEQNNPHTPDYEFVTAKPGSSMTIACTYDGIVSAIYHVIMQTNGLIYASFMNGACGSVGYTLKNGKLLLHYMHHLE
FNNKTHGGTDLNGSFYGDYIDEEIAQSISKAATLTDNALAHIYAHLSTISTKPKWLSYQELSVEDFNEWAKNNDHTQFPSCDE
NYTYLDALAKSTGVSITRALSTLVTLHTNWGSASVLGMSTFDLDFTPEMVYNQAPITLQ

>Quail coronavirus UAE-HKU30
AGIKILLHPSGVVERCIVSVVYNGSALNGIWLNNVVYCPRHVIGKYRGEQWSHMVSIADCREFIVKCPTQGVQLNVQSVKMVG
ALLQLTVHTNNTATPNYKFERLAPGSSMTIACAYDGVVRHVYHVVLQTNYLIYASFLNGACGSVGYTLKGKTLCLHYMHHIEF
NNKTHSGTDLEGNFYGPYVDEEVVQHQTAFQYYTDNVVAQLYAHLLTVDARPKWLAHAQISVDDFNAWAANNSFASFPCEQTN
MSYIMGLSQTARIPVERILNTIVQLTLNRDGAIIMGSPDFECDWTPEMVYNQ

>Bulbul coronavirus HKU11-796
AGIKILLHPSGVVERCMVAVTYNGSALNGVWLNNVVYCPRHVLGKYRGEQWQHMVSIADCRDFAITCPAQGIQLTVQSIKMVG
AILQLTVHTSNSGTPDYEFVRITPGSSMTIACAYDGVVRNVYHVVLQTNNLIYASFLNGACGSVGYTLRGKTLLLHYMHHLEF
NNQTHGGTDLHGQFYGPYVDEEVAQQQTAFQYYTDNVVAQLYAHLLTIDASPRWLASAEISESDFNAWASSNSFANFPCEQSN
MAYILGLSQTAKVSVGRILNTIIQLTLNRSGALIMGKPDFECDWTPEMVYNQAPITLQ

Figure S2. 3CLpro sequences of different coronavirus lineages. A search was conducted using BLASTP to find representatives of each genus, excluding SARS-CoV-2 3CLpro sequences at all stages. This search resulted in sixteen sequences from different coronavirus lineages

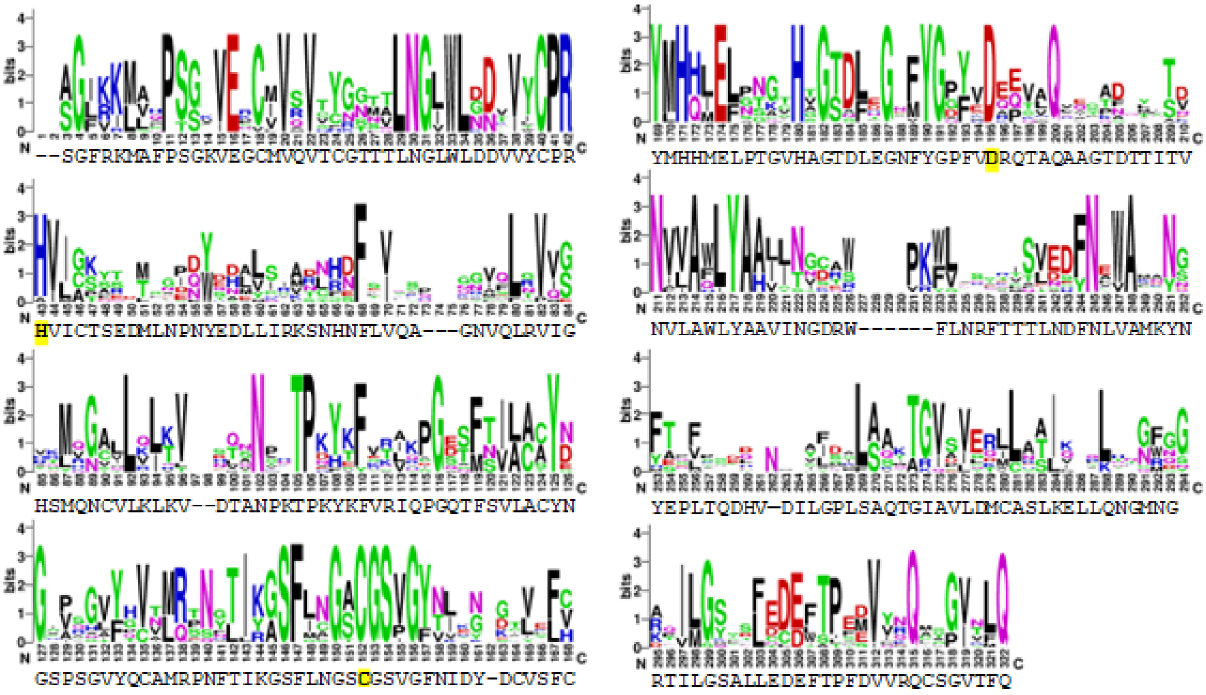

Figure S3. Sequence logo. The sequence logo representing the multiple sequence alignment shows that the His41-Cys145-Asp187 triad is absolutely conserved.

Table S1. The results of the search for structural homologues of 3CLpro in human proteins, obtained from the Dali server. PDB ID, which identifies protein structures, the Z-score, indicating structural similarity, the RMSD to measure discrepancies between structures, the ID (%) to evaluate the similarity of amino acid sequences and the name of the protein under analysis.

PDB ID	Z-score	RMSD	ID (%)	Protein
8s9k	11.5	3.3	16	SERINE PROTEASE FAM111A
5to3	10.5	2.9	10	PROTHROMBIN
1z8g	10.4	2.9	16	SERINE PROTEASE HEPSIN
4kkd	10.1	3.4	11	MANNAN-BINDING LECTIN SERINE PROTEASE
2xwb	10.1	3.2	8	COMPLEMENT C3B BETA CHAIN
7wqx	9.7	3.5	9	ENTEROPEPTIDASE
3h5c	9.6	2.7	12	PROTEIN Z-DEPENDENT PROTEASE INHIBITOR
2xrc	9.6	3.1	14	HUMAN COMPLEMENT FACTOR I
5i25	9.4	2.8	9	COAGULATION FACTOR XI
4a5t	9.1	3.1	8	PLASMINOGEN
